# Research Evaluating Staff Training Online for Resilience (RESTORE):  Protocol for a single-arm feasibility study of an online Acceptance and Commitment Therapy intervention to improve staff wellbeing in palliative care settings

**DOI:** 10.12688/amrcopenres.13035.1

**Published:** 2021-11-18

**Authors:** Anne Finucane, Nicholas J Hulbert-Williams, Brooke Swash, Juliet A Spiller, Brigid Lydon, David Gillanders

**Affiliations:** 1 Clinical Psychology, University of Edinburgh, Edinburgh, UK; 2 Marie Curie, Edinburgh, UK; 3 School of Psychology, University of Chester, Chester, UK

**Keywords:** Acceptance and Commitment Therapy, behaviour therapy, cognitive behavioural therapy, palliative care, hospice and palliative care nursing, wellbeing, stress, burnout

## Abstract

**Background:**

Palliative care workers commonly experience workplace stress and distress. General stressors include unmanageable workloads and staff shortages. Stressors specific to palliative care include regular exposure to death, loss and grief. The COVID pandemic exacerbated exhaustion and burnout across the healthcare system, including for those providing palliative care. Evidence based psychological support interventions, tailored to the needs and context of palliative care workers, are needed. Acceptance and Commitment Therapy (ACT) is an established form of cognitive behavioural therapy which uses behavioural psychology, values, acceptance, and mindfulness techniques to improve mental health and wellbeing. ACT is effective in improving workplace wellbeing in many occupational settings. Our study examines the acceptability and feasibility of an online ACT-based intervention to improve mental health and wellbeing in staff caring for people with an advanced progressive illness.

**Methods:**

A single-arm feasibility trial. We will seek to recruit 30 participants to take part in an 8- week online ACT-based intervention, consisting of three synchronous facilitated group sessions and five asynchronous self-directed learning modules. We will use convergent mixed methods to evaluate the feasibility of the intervention. Quantitative feasibility outcomes will include participant recruitment and retention rates, alongside completion rates of measures assessing stress, quality of life, wellbeing, and psychological flexibility. Focus groups and interviews will explore participant perspectives on the intervention. We will run a stakeholder workshop to further refine the intervention and identify outcomes for use in a future evaluation.

We will describe participant perspectives on intervention acceptability, format, content, and perceived impact alongside rates of intervention recruitment, retention, and outcome measure completion.

**Conclusion:**

We will show whether a brief, online ACT intervention is acceptable to, and feasible for palliative care workers. Findings will be used to further refine the intervention and provide essential information on outcome assessment prior to a full-scale evaluation.

## Plain language summary

Palliative care workers commonly experience workplace stress and distress. General stressors include unmanageable workloads and staff shortages. Stressors specific to palliative care include regular exposure to death, loss and grief. The COVID pandemic increased exhaustion and burnout across the healthcare system, including for workers providing palliative care. An introduction to psychological therapies for professionals caring for people with a terminal illness may improve workplace wellbeing. Acceptance and Commitment Therapy (ACT) is an established form of cognitive behavioural therapy which uses behavioural psychology, values, acceptance, and mindfulness techniques to improve mental health and wellbeing. ACT is effective in improving workplace wellbeing in many occupational settings though has not yet been evaluated with hospice staff. Our study will examines whether a brief ACT intervention, delivered online, is helpful to hospice staff, and research on this can be carried out.

We will seek to recruit 30 hospice staff participants to take part in an 8-week online ACT-based intervention, consisting of three online in-person group sessions and five self-directed learning modules. We will gather date from a range of sources, including questionnaires and focus groups, to work out whether there is value in this type of intervention.

We will describe participant views on the intervention, what worked and what needs to be refined; alongside data on the number of people interested in participating and of those the proportion completing the intervention.

We will show whether a brief, online ACT intervention can potentially improve wellbeing for palliative care workers, and can be evaluated in future studies. Findings will be used to further refine the intervention and provide essential information for future research.

## Introduction

Healthcare professionals working in palliative care settings commonly experience stress and distress ^1– 3^. General stresses include unmanageable workload and staff shortages. Specific stressors occur as a result of caring for patients with complex physical conditions, and regular exposure to death, loss, and grief. In 2020-21, the COVID pandemic led to increased stress and distress within the health and social care workforce ^4– 8^, including palliative care ^9, 10^. As a result of the pandemic, healthcare workers encountered rapidly changing clinical roles, new modes of service delivery and increased patient volumes; coupled with risk of infection and limitations in personal protective equipment. A considerable proportion experienced mood and sleep disturbances, raising concerns about risks to mental health ^8^. 

Despite the occurrence of workplace stress and distress, evidence based psychological support for palliative care workers is lacking. A 2019 Nursing Standard-Marie Curie survey involving 5,346 UK nurses and healthcare assistants involved in end-of-life care, found that one-third reported insufficient support at work to manage grief and emotional stress from caring for dying patients ^11^. In 2020, the same survey with 894 respondents, revealed this proportion had increased to 45%, with visiting restrictions due to the pandemic placing additional emotional burden on staff who needed to balance the safety of patients with their needs to be together with their families ^12^. A systematic review prior to the pandemic identified only nine papers evaluating psychosocial interventions for palliative care staff and concluded that there was an urgent need to address the lack of intervention development work and high-quality research in this area ^13^. Flexible, accessible, scalable and cost-effective psychological support interventions are now more important than ever. 

We propose an Acceptance and Commitment Therapy (ACT) based psychological intervention to improve wellbeing, stress, and distress in palliative care workers. ACT is an established form of cognitive behavioural therapy which uses behavioural psychology, values, acceptance and mindfulness techniques to improve mental health and wellbeing ^14^.

ACT principles target the kinds of responses that clinicians identify as helpful: being present, finding purpose, acceptance, perspective taking and engaging in life ^15^. Recent systematic reviews and meta-analyses show that ACT is efficacious in treating stress, anxiety and depression in a range of settings ^16– 18^, including when delivered online ^19, 20^. Drawing on evidence of effectiveness in other occupational and healthcare settings ^16, 17, 21– 23^, we propose that an ACT-based psychological intervention has strong potential to improve mental health and wellbeing in palliative care workers.

We aim to develop, and feasibility test an online ACT-based intervention to enhance workplace wellbeing of staff working with terminally ill and dying patients, and their families. Our research will answer the following questions:

- Is an online ACT intervention feasible and acceptable to palliative care workers?
- What is the experience of palliative care staff undertaking ACT training?
- What are the barriers and facilitators to implementing an online ACT intervention for palliative care workers?
- Is there preliminary evidence that ACT training leads to improvements in workplace wellbeing and stress in palliative care staff?
- What are the implications for future evaluation research, including sample size and outcome measures?

## Methods

### Design

A single-arm feasibility trial of a brief ACT-based intervention for staff providing palliative care for terminally ill adults. We will use convergent mixed methods ^24^ to evaluate the feasibility of the proposed intervention. 

### Setting

The study will be hosted by Marie Curie Scotland. Marie Curie is the largest independent provider of end-of-life care and the largest charitable provider of hospice-based care in Scotland. Two Marie Curie hospices, located in Edinburgh and Glasgow, provide short-term inpatient hospice care, outpatient services (including day services), home visits and family support, to terminally ill people and families in their surrounding areas. Across Scotland, the Marie Curie Nursing Service (MCNS) provide care in the last days of life to people in their own homes.

### Participants and sample size

Participants will be recruited from two Marie Curie hospices and from the Marie Curie Nursing Service (Scotland). We will seek a sample size of approximately 30 participants. As this is a feasibility study, sample size has not been formally calculated. Our target sample size is based on prior experience, resources available and the format of the intervention. 

***Inclusion criteria.*** The intervention will be offered to health and social care professionals providing direct support to patients and families, including nursing and medical staff, allied health professionals, social workers, and healthcare assistants. 

***Exclusion criteria.*** Staff who have previously undertaken Acceptance and Commitment Therapy training in the past.

### Participant recruitment

Members of the research team will discuss the proposed study with service managers and clinical leads across Marie Curie Scotland. The study will be promoted via hospice newsletters, posters, internal email lists and via relevant Marie Curie internal online forums. The research team will run an online information session for potentially interested participants via MS Teams. 

Interested staff members will be directed to seek approval from their line manager in the first instance if they are interested in taking part. Potentially interested participants will be emailed the participant information sheet, and a link to an online consent form. Following consent, they will be invited to complete an online ‘participant characteristics form (gender; age band; ethnicity; role and number of years’ experience in palliative care) which will be used to describe the sample, and stored separately from any outcome data.

### Intervention design and content

***Location.*** The online Acceptance and Commitment Therapy (ACT) intervention will be hosted using MS Teams. Participants will be able to join from their usual workplace or home setting. Support will be provided from the research team and the admin team at each hospice for participants who need to access a hospice PC in a quiet location for the purpose of this study.

***Facilitation.*** The intervention will be delivered by a Peer Reviewed ACT Trainer and Fellow of the Association for Contextual Behavioural Science (DG) and facilitated by a research psychologist (AF). DG will lead the virtual classroom sessions. AF will co-ordinate module delivery, manage the online platform and liaise and respond to technical support queries.

***Online platform.*** MS Teams will be used as the training platform, as this supports a range of media, enables chat, and is used widely within health and social care organisations. An MS Team will be set up for intervention delivery, and participants will be invited to join once they consent to the taking part. All participants will be invited to access the online platform in advance of the first session, and individual support, as well as printed resources, will be provided to support access. All intervention materials made available via MS Teams. 

***Content.*** The intervention will cover key ACT processes including being present, finding purpose, perspective taking and engaging in life values ( Table 1). The content has been informed by previous interventions delivered by the team for health professionals in other settings, as well as research on occupational stress, wellbeing, and resilience in palliative care. The format is described in a draft manual, which will be revised and shared on the ISRCTN registry on completion of the study.

**Table 1.** Overview of intervention content.

Week	Module	Time required	Content	Delivery mode
*1*	***Introduction to ACT***	**90 minutes**	**Introduction**	Synchronous online virtual classroom Led by an ACT trainer (DG)
*2*	***Values***	30 minutes	Identifying and acting in line with your values	Self-directed, asynchronous materials Group chat Homework
*3*	**Awareness**	30 minutes	Present moment awareness; mindfulness; grounding	Self-directed, asynchronous materials Group chat Homework
*4*	***Review of materials***	**90 minutes**	Review, troubleshooting, clarifying materials. Discussion.	Synchronous online virtual classroom Led by an ACT trainer (DG)
*5*	***Openness***	30 minutes	Developing self-awareness, becoming more open, making room.	Self-directed, asynchronous materials Group chat Homework
*6*	***Defusion***	30 minutes	Unhooking from difficult thoughts and feelings	Self-directed, asynchronous materials Group chat Homework
**7**	**Compassion**	30 minutes	Kindness to self and others	Self-directed, asynchronous materials Group chat Homework
**8**	**Review and** **trouble shooting**	**90 minutes**	Review, troubleshooting, clarifying materials. Discussion.	Synchronous online virtual classroom Led by an ACT trainer (DG)

***Format.*** There will be 8 modules delivered via MS Teams over an 8-week period ( Table 1). Delivery will be via three synchronous virtual classroom sessions and five asynchronous, self-directed, e-learning modules. The three virtual classroom modules will be interactive expert-led sessions. The five self-directed e-learning modules will consist of online reading materials, pre-recorded videos, and reflective exercises. Participants will be encouraged to share experiences with each other and ask questions or seek clarification via the online chat function within MS Teams. Participants will be provided with a workbook (hard-copy) outlining the content for each week alongside related exercises. 

***Homework.*** Participants will be asked to complete ‘homework’ each week to reflect on the material and how it relates to themselves and their work. Homework will be brief and non-obligatory but encouraged. Examples of homework would include, for example, practicing brief mindfulness exercises or exercises to enhance compassionate responses. Homework will be described in the workbook. 

***Adherence*** To facilitate adherence, participants will also be sent a weekly reminder to alert them when new content is made available. To facilitate engagement participants will be encouraged to share their experiences of the intervention, and any questions they might have via the online chat function (within MS Teams). 

***Benefits of participation.*** Participants will receive introductory training in ACT targeted at using this for their own stress management benefit and will be given the opportunity to engage with a range of strategies to improve their workplace and personal well-being over time. 

***Distress protocol.*** It is unlikely that a participant will experience negative consequences as a result of participating in the proposed intervention. However, if a participant does report feeling heightened stress or distress, the course leader, a Clinical Psychologist, will discuss immediate concerns with them, and will signpost them to additional resources and support as appropriate. This may include: i) the Marie Curie Employee Assistance Programme (EAP) which includes practical information and emotional support guidance as well as access to trained counsellors; ii) to their line manager or HR manager if appropriate; iii) to a Marie Curie hospice-based Clinical Psychologist. Any unintended harms will be recorded by the Co-Principal Investigators and described as part of the research findings.

***Ancillary and post-trial care.*** Should any participant express a need for additional or continued psychological support on completion of the intervention, they will be directed to additional support in line with the distress protocol.

### Data collection, analysis and management

***Quantitative data collection.*** Five key outcomes will be assessed prospectively, via JISC online survey ( www.onlinesurveys.ac.uk) at four time-points – before commencement of the intervention, mid-way through the intervention, on completion of the intervention and one-month post-completion. ( Table 2). This will allow examination of preliminary evidence for changes in outcomes over the course of the intervention, as well as outcome completion rates to inform the design of a future evaluation. As we are most interested in changes in outcomes that are sustained beyond intervention delivery, our main exploratory analysis will focus on the change in outcomes pre-intervention versus 4 weeks post intervention. This data will help us identify key outcomes and sample size needed for a future evaluation

**Table 2.** Schedule of enrolment, interventions, and assessments.

WEEK	Pre-intervention	Wk1	Wk2	Wk3	Wk4	Wk5	Wk6	Wk7	Wk8	Wk9	Wk10	Wk11	Wk12	Wk13-16
**EVALUATION** **TIMEPOINTS**	**t0**			**t1**				**t2**					**t3**	
**ENROLMENT**														
Study promotion	X													
Informed consent	X													
Technology support session	X													
**INTERVENTION**		Introduction	Values	Awareness	Review	Openess	Diffusion	Compassion	Review					
Virutal classroom modules		X			X				X					
E-Learning module			X	X		X	X	X						
**QUANTITATIVE** **OUTCOMES**														
Perceived stress	X			X				X					X	
Professional Quality of Life	X			X				X					X	
Mental wellbeing	X			X				X					X	
Mindful Healthcare	X			X				X					X	
Psychological flexibility	X			X				X					X	
Feasibility data collection										X				X
**QUALITATIVE** **DATA**														X
Focus groups														X
Interviews														X

***Self-reported, perceived stress*** will be assessed by the Perceived Stress Scale (PSS) ^25, 26^. This scale assesses participants' appraisals of stressful situations, including perceptions of how unpredictable, uncontrollable, and overloaded their lives have been over a defined period. We will use the 10-item scale consisting of a Perceived Helplessness subscale (6-items) and a Perceived self-efficacy subscale (4-items) ^26^.

***Workplace quality of life*** will be assessed by the Professional Quality of Life scale (ProQol) ^27^. The ProQOL consists of 30 items in three subscales (10 items per subscale) designed to measure *compassion*, *satisfaction*, *burnout*, and *compassion fatigue*. Each item rates the frequency of an experience on a scale from 0 (never) to 5 (very often).

***Wellbeing*** will be assessed using the Edinburgh Warwick Mental Wellbeing Scale ^28, 29^. This is a 14-item scale, overing both hedonic and eudemonic aspects of mental health including positive affect, satisfying interpersonal relationships and positive functioning. Higher scores reflect increased wellbeing.

***Healthcare professional experience*** will be assessed by the Mindful Healthcare Scale which assesses engagement, awareness and defusion – 13 items. This measure is being developed at the University of Edinburgh ^3, 30^.

***Psychological flexibility*** will be assessed by the CompACT. ^31^ It consists of 23 items over 3 sub-scales - *openness to experience*, *behavioural awareness*, *and valued action*. Participants are asked to rate the degree to which a statement is true for them using a 7-point scale. The higher the score, the higher participant's level of psychological flexibility.

**Feasibility outcomes** will be assessed retrospectively via routine data collection on completion of the intervention and qualitative data collection. Data will include:

- the number of participants recruited (target is 24 – 30 participants),
- the number of participants who complete the intervention, i.e. participant attended the final session or reported they had completed at least 6 of 8 modules (target is two-thirds of those recruited)
- the number of participants who complete the outcome measures (target is at least two-thirds of those who commence the intervention).
- The number of participants who take part in post-intervention focus groups or interviews (target is 50–75% of those recruited given that focus groups will be run during work time and some participants will be unable to take part due to work schedules and annual leave).

We will also explore stress levels at baseline to determine whether those with moderate to high levels of stress were recruited and examine any potential links between baseline stress and participant retention.

***Qualitative data collection.*** We will conduct virtual focus groups with participants one month following completion of the intervention to explore their experience of ACT training; views on what elements were most and least useful; perception of any changes in how they manage challenging situations; and whether/in what way skills learnt during the training might improve the care they provide to patients and families. All participants will be invited to take part. Focus groups will be run via MS Teams, recorded, and transcribed for analysis. Qualitative data will be collected by an experience qualitative researcher (BS), based at a different institution to participants, and not personally known to participants prior to data collection. Participants who drop-out will be invited to share their reasons for drop-out brief interview. With permission, the interview will take place via MS Teams, and will be recorded and transcribed. Where participants drop-out and choose not to take part in an interview, we will ask permission to note their main reason(s) for drop-out.

***Quantitative and qualitative data analysis.*** The main quantitative outcomes of interest are feasibility outcomes which will include rates of participant recruitment and retention over the course of the study. As this is a feasibility trial, it is likely to be underpowered to detect statistically significant improvements in the non-feasibility outcomes (e.g. stress, quality of life, wellbeing) and so these will be reported descriptively (mean, standard deviation, range etc.). Instead, the outcome data collected will help ascertain whether there is any preliminary evidence for intervention effectiveness. It will be also used to estimate effect sizes and determine sample sizes for a future full-scale evaluation. Any missing data will be handled using pairwise deletion methods. We are most interested in any evidence for improvements in outcomes between pre-intervention baseline (T0) and post-intervention follow-up (T3). Data will be analysed in IBM SPSS Statistics, V24. Qualitative data analysis will be guided by the framework approach using NVivo 12 ^32^ and will provide vital insights on intervention acceptability, perceived effectiveness and recommendations for further refinement.

***Stakeholder workshop to cross-validate emergent findings, refine the intervention and identify considerations for future full scale evaluation and implementation.*** A stakeholder workshop will be organised, bringing together participants, members of the research team, staff managers and those who would be involved in intervention delivery if it were implemented to discuss findings. At this workshop participants will have the opportunity to highlight themes that resonate with them, as well as those that do not, and identify anything that may have been missed. We will review feasibility data and seek feedback on how the intervention could be further refined to meet the needs of palliative care staff in the organisational context in which the intervention would be implemented. We will explore costs and resource use from multiple perspectives and consider short and long terms outcomes for consideration in a future full-scale evaluation. 

### Data management

***Personal data.*** Personal data (participant name, email address, age band, gender, role, years working in palliative care) will be stored in a secure file in the University of Edinburgh DataStore. This is password protected and encrypted storage. Participants will be allocated a Participant ID for the study. The Participant ID will be used when completing the online questionnaire. Outcome data will be kept separate from personal data throughout the study. The code break file will be held in a separate password protected folder within the DataStore only accessible by the Co-Principal Investigators (AF and DG). 

***Transfer of data.*** Focus group and interview data collected at the host organisation (Marie Curie) will be stored in a secure location on MS Teams, accessible only to participants and the research team (and deleted once transcription has taken place). Data will be anonymised and transcribed by a member of the research team, and then transferred via secure email to the University of Edinburgh DataStore. 

***Data storage.*** All date files will be stored will be stored in a secure location – the University of Edinburgh DataStore. Personal data will be held for no longer than 2 years after completion of the study. The Co-Principal Investigators will be responsible for deletion of data. Anonymised data will be retained indefinitely to inform future research.

***Confidentially.*** Participants will be allocated a Participant ID following recruitment. The Participant ID will be used when completing online questionnaires throughout the study. The Participant ID will be linked in one stand-alone file held in the DataStore, and only accessed if the participant has forgotten their ID and needs to be reminded. We do not plan any analysis that would potentially identify a participant- however, if there is any chance of this occurring, we will collapse the small group data in the final anonymised file, so there is no chance of identification (e.g. If only one male participated, outcome data by gender would be amalgamated.) Participants will be known to each other but will be reminded throughout the study that all discussions should remain confidential.

### Monitoring

***Role of sponsor and funder.*** The Co-Principal Investigators (AF and DG) are employees of the sponsor organisation (University of Edinburgh) and have honorary contracts at the host site (Marie Curie). They Co-Principal Investigators will have a direct role in study design; collection, management, analysis, interpretation of data; writing of the report; and the decision to submit the report for publication. The study is funded by Marie Curie: www.mariecurie.org.uk.

***Data controller, breaches and monitoring.*** The University of Edinburgh is the data controller for this study. Any data breaches will be reported to the University of Edinburgh Data Protection Officers who will onward report to Marie Curie, according to the appropriate timelines, if required. As this is a low-risk single-arm feasibility study, a data monitoring committee is not required.

## Ethics and dissemination

***Research ethics and governance approvals.*** We obtained ethical approval from the University of Edinburgh Clinical Psychology Research Ethics Committee on 20/5/2021 (Ref: CLPS021s). This research was also approved by the Marie Curie Research Governance Committee in Scotland.

***Protocol registration and amendments.*** Any protocol amendments will be communicated to i) the sponsor, ii) the University of Edinburgh Clinical Psychology Research Ethics Committee, and iii) the Scottish Marie Curie Research Governance Committee, in a timely manner. The study protocol was registered on the ISRCTN registry, and any protocol amendments will be available there ( https://doi.org/10.1186/ISRCTN14313559). 

### Dissemination policy

Research findings will be disseminated via publication in an open access academic journal, a report for the funder, social media and conference presentation. To accelerate dissemination, we will share study findings on a pre-print server (e.g. Medrxiv) at the same time as we submit to a journal. We will share preliminary findings (e.g. poster presentations) and related study material (e.g. study protocol) on AMRC Open ( https://www.amrc.org.uk/), an open access platform for the dissemination of research funded by medical research charities.

Authorship of research papers will be agreed in line with ICMJE recommendations. http://www.icmje.org/icmje-recommendations.pdf. 

## Conclusions

The RESTORE study represents the first step in a programme of research to examine the effectiveness of ACT-based approaches to improve mental health and wellbeing in health and social care professionals caring for people with an advanced illness. It will yield findings on the acceptability and feasibility of an online ACT intervention for palliative care workers, including the aspects of the intervention most valued, and the outcomes most useful to measure in future studies. Our findings will inform the development of a future large scale evaluation examining intervention effectiveness on staff mental health and wellbeing, cost effectiveness and broader impacts within the broader health and social care system. 

### Study status

Data collection is currently underway, and will be completed in December 2021. Data analysis will take place in January 2022, and the stakeholder workshop will take place soon after. The study is scheduled to conclude in March 2022.

## Data availability

The Co-Principal Investigators and Co-Investigators will have access to the final dataset. On completion of this study fully anonymised underlying data will be made available on the ISRCTN registry. 

### Reporting guidelines

The SPIRIT reporting guideline was used in writing this protocol and is available here: https://doi.org/10.1186/ISRCTN14313559.
