# Supplementary material for: Research Evaluating Staff Training Online for Resilience (RESTORE):  Protocol for a single-arm feasibility study of an online Acceptance and Commitment Therapy intervention to improve staff wellbeing in palliative care settings
Source: AMRC Open Res. 2022 Jun 22;3:26. Originally published 2021 Nov 18. [Version 2] doi: 10.12688/amrcopenres.13035.2 (PMC11064981; doi:10.12688/amrcopenres.13035.2)
Supplement: Peer review history

# Reviewer report: Reviewer response for version 2

**DOI:** 10.21956/amrcopenres.14151.r26973

**Published:** 2022-07-11

**Authors:** David Dawson

**Affiliations:** 1 School of Psychology, University of Lincoln, Lincoln, UK

The authors have addressed the comments provided in the original review.

Is the study design appropriate for the research question?

Partly

Is the rationale for, and objectives of, the study clearly described?

Yes

Are sufficient details of the methods provided to allow replication by others?

Yes

Are the datasets clearly presented in a useable and accessible format?

Partly

Reviewer Expertise:

Clinical Psychology; Applied Clinical Research; Behaviour Analysis; Behaviour Change

I confirm that I have read this submission and believe that I have an appropriate level of expertise to confirm that it is of an acceptable scientific standard.

# Reviewer report: Reviewer response for version 2

**DOI:** 10.21956/amrcopenres.14151.r26972

**Published:** 2022-06-23

**Authors:** Jason Mills

**Affiliations:** 1 School of Nursing, Midwifery and Paramedicine, University of the Sunshine Coast, Caboolture, Qld, Australia

Careful consideration of feedback is evident and the authors have revised this paper to a very high standard.

Is the study design appropriate for the research question?

Yes

Is the rationale for, and objectives of, the study clearly described?

Partly

Are sufficient details of the methods provided to allow replication by others?

Partly

Are the datasets clearly presented in a useable and accessible format?

Yes

Reviewer Expertise:

Palliative care, Mindfulness, Compassion, Self-compassion, Self-care, Workforce

I confirm that I have read this submission and believe that I have an appropriate level of expertise to confirm that it is of an acceptable scientific standard.

# Reviewer report: Reviewer response for version 1

**DOI:** 10.21956/amrcopenres.14110.r26967

**Published:** 2022-05-19

**Authors:** David Dawson

**Affiliations:** 1 School of Psychology, University of Lincoln, Lincoln, UK

Thank you for the opportunity to review this study protocol which outlines a single-arm feasibility study of an online Acceptance and Commitment Therapy (ACT) intervention to improve staff wellbeing in palliative care settings.

 The authors provide a clear rationale and justification for the proposed work: the elevated levels of stress and distress among palliative care workers, the augmenting effect of the COVID-19 pandemic on those stressors, and the lack of evidence-based interventions available to palliative care workers to mitigate the psychological impact of their work.  

 The work has clear merit and has the potential to significantly impact on clinical practice; successful outcomes from the work have implications for practitioners (potentially including improved psychological health, and reduced stress, burn-out, and compassion fatigue), services (perhaps improved staff retention, lower levels of absence), and service-users (improved and more engaged care). The work also has the potential to contribute to the extant theoretical literature within behavioural health science.

 The protocol is generally well-written, clearly structured, and appropriately detailed; I have provided some comments below that largely focus on the methodological aspects of the work, and hope the authors find them constructive – either for informing the current work, or a subsequent full trial.

- Inclusion criteria: although the primary focus of the work at this stage is feasibility, it would perhaps be beneficial to state a preliminary primary outcome (e.g., Perceived Stress, Wellbeing), and to only recruit participants to the study who already meet a minimum threshold on that measure (e.g., ‘moderate stress’). That would help protect against floor/ceiling effects, ensure that any meaningful change within those variables was observable/captured by the measures, and potentially improve the ability to detect signal efficacy.

- Design: The rationale for the non-randomised, single-arm design, and the decision to not include a ‘wait-list’ or similar control (to facilitate randomisation, control for within participant differences, and establish between group effects) was not clear and would be useful to include.

- Fidelity: Some detail regarding how fidelity to the ACT model (within the online sessions) is to be assessed would be useful (e.g., independent review of online seminars using the ACT-FM). This is particularly salient given that session seven within the protocol suggests a focus on ‘Compassion’ (which may adhere more closely to Compassion-Focused Therapy rather than traditional ACT).

- Measures: To lessen participant burden, the authors may wish to consider the short versions of the WEMWBS and CompACT.

- Adherence: Will homework compliance etc., be formally monitored (to determine adherence to, and ‘dose’ of, the intervention)?

- Data collection: The authors may not be aware that the JISC platform (at the time of writing) does not appear to allow analysis of partial or incomplete responses ( https://www.onlinesurveys.ac.uk/help-support/can-i-see-partial-or-incomplete-responses/). This appears to be a severe limitation of the platform, particularly when undertaking feasibility research. The authors state here their aim to determine the number of participants who complete the measures; however, partial, incomplete, and unfinished survey responses cannot be accessed – only the page where a participant ceased participation can be determined.

- It would be useful to provide a rationale for the use of focus groups (over individual interviews) given the impact dominant voices can have on group consensus etc.

- Stakeholder Workshop: We have found the RE-AIM framework useful for structuring discussions around how health interventions might be refined ( https://re-aim.org/learn/what-is-re-aim/) and the authors may find some utility there too.

Is the study design appropriate for the research question?

Partly

Is the rationale for, and objectives of, the study clearly described?

Yes

Are sufficient details of the methods provided to allow replication by others?

Yes

Are the datasets clearly presented in a useable and accessible format?

Partly

Reviewer Expertise:

Clinical Psychology; Applied Clinical Research; Behaviour Analysis; Behaviour Change

I confirm that I have read this submission and believe that I have an appropriate level of expertise to confirm that it is of an acceptable scientific standard.

# Response

**Published:** 2022-05-31

**Authors:** Anne Finucane

**Affiliations:** Marie Curie Cancer Care, UK

Thank you very much for the helpful comments and suggestions. These will be very informative as we plan follow-up work.  We have provide some additional justifications and clarifications in response to your comments below. 

1. We did not specify a primary outcome or pre-screen participants as this was a small-scale feasibility study. We were keen to explore a range of outcomes to see which might be most suitable for inclusion in future large-scale evaluations. We were also unsure about the extent to which would be able to recruit participants in light of COVID, so were hesitant to pre-screen participants, but we will explore the question of whether or not to pre-screen in future studies as we analyse the data collected and share the findings with stakeholders.
2. As this was a small-scale study, undertaken during the COVID pandemic, we were keen to keep it simple. We did not have the capacity to link in with clinical trials unit, and to randomise participants. However, we agree with Reviewer 2 that this will be essential in future studies.
3. Thank for this useful suggestion which we will need to consider for future studies. For the present study, the intervention was delivered by a Peer Reviewed ACT Trainer and Fellow of the Association for Contextual Behavioural Science, but for future studies we will need to consider fidelity assessments.
4. We aimed to collect data via focus groups as this method allows the generation of a range of responses regarding the intervention in an efficient and pragmatic manner. Furthermore, participants were used to exchanging views in a facilitated group format, and while we appreciate that in some instances this can hinder the freedom with people exchange views, given the nature of the topics covered in the focus groups, it was felt that the benefits of the group interaction and sharing of ideas outweighed this. We also offered interviews to participants who did not complete the intervention. Given the timing of intervention delivery (during the height of the COVID pandemic) offering a variety of participation approaches, while minimising overall staff time commitment, was important. 
5. Thank you for the helpful suggestion in relation to using shorter assessment tools which we will consider in future studies.
6. Adherence was monitored via self-report questionnaires and explored further during qualitative data collection.
7. We set up 4 separate questionnaires to gather data at each timepoint; and merge on completion of data collection (using participant ID not linked to the participants name) - any limitations of the JISC platform have not been a problem.
8. Thank you for the helpful suggestion in relation to the RE-Aim framework.

# Reviewer report: Reviewer response for version 1

**DOI:** 10.21956/amrcopenres.14110.r26868

**Published:** 2021-12-15

**Authors:** Jason Mills

**Affiliations:** 1 School of Nursing, Midwifery and Paramedicine, University of the Sunshine Coast, Caboolture, Qld, Australia

This article reports the RESTORE study protocol - Research Evaluating Staff Training Online for Resilience (RESTORE) – seeking to examine the feasibility and acceptability of an Acceptance and Commitment Therapy (ACT) intervention designed to improve mental health and wellbeing for those working within palliative care contexts.

 I commend the researchers on their efforts in this important area.

 Based on my experience as an ACT practitioner, as well as my knowledge and understanding of the palliative care field, I believe this paper proposes an important study of considerable merit given the past and ongoing impacts of the COVID-19 pandemic on the study population.

 Overall, the rationale and objectives of the study are mostly described in a clear way. However, from my review, there are some important considerations for minor revision and the future conduct/reporting of the study. These are outlined further below.

 This single-arm trial incorporating a mixed-methods convergent design is appropriate for the broad research questions being addressed in this study, with the mixing of quantitative and qualitative data representing a strength. Consideration of improving consistency in research question nomenclature is recommended (the terms ‘palliative care workers’ and ‘palliative care staff’ are used interchangeably).

 Details of research methods are sufficient for a protocol at this stage, although there is scope to strengthen the reporting of the intervention content/format when finalising the ‘draft manual’ prior to sharing on the ISRCTN registry.

 How was the content developed and how is the content/format justified? These details appear to be missing from the protocol. On p.4 the reader is informed that ‘the content has been informed by previous interventions delivered by the team for health professionals in other settings, as well as research on occupational stress, wellbeing, and resilience in palliative care’ – however, there are no intervention reference sources or research reports cited in this sentence to substantiate or support this claim.

 To this end, there is a need to draw more direct linkage between self-compassion and the ACT intervention.

 While it is pleasing to see the inclusion of intervention content focused on compassion for self and others (Module 7), from my review this content has not been adequately justified and/or situated within the context of those working in palliative care. The relevance and remit of self-compassion in hospice and palliative care has been systematically reviewed by Garcia et al (2021) ^1^.

 Given the focus of module 7 and the homework exercises intended to ‘enhance compassionate responses’ in participants, it is recommended that the authors include some reference to the relevant literature to justify/better situate this important content as part of the intervention. This is pertinent since that module is self-directed rather than facilitated, and past research in this area has indicated compassion for self does not come naturally and can be challenging for some people working in palliative care. The Module 8 ‘Review and trouble-shooting’ session might best begin with acknowledging this known challenge and inviting discussion of any challenges/barriers experienced.

 Important correction required:

- There is an error requiring correction on p.5 (Data collection, analysis and management) – under the heading of ‘Workplace quality of life’ it is stated that ‘The ProQOL consists of 30 items in three subscales (10 items per subscale) designed to measure compassion, satisfaction, burnout, and compassion fatigue’. However, this instrument does not measure compassion and it was not designed to do so. Apart from BO and CF, the ProQOL measures ‘compassion satisfaction’ (CS) which is quite distinct from compassion itself. Perhaps this was a typo – with an erroneous comma in between ‘compassion’ and ‘satisfaction’?  Please correct this sentence for veracity.

Recommended minor revisions:

1. As above, please consider drawing reference to the relevant literature on self-compassion in palliative care to justify and better situate its explicit inclusion as key content (Module 7) within the ACT intervention. See for example: Garcia et al. (2021). https://journals.lww.com/jhpn/Abstract/2021/04000/Self_compassion_In_Hospice_and_Palliative_Care__A.8.aspx ^1^.
2. Consider improving consistency in the nomenclature used in the stated research questions (the terms ‘palliative care workers’ and ‘palliative care staff’ are used interchangeably).

Very best wishes for progressing this work.

Is the study design appropriate for the research question?

Yes

Is the rationale for, and objectives of, the study clearly described?

Partly

Are sufficient details of the methods provided to allow replication by others?

Partly

Are the datasets clearly presented in a useable and accessible format?

Yes

Reviewer Expertise:

Palliative care, Mindfulness, Compassion, Self-compassion, Self-care, Workforce

I confirm that I have read this submission and believe that I have an appropriate level of expertise to confirm that it is of an acceptable scientific standard, however I have significant reservations, as outlined above.

## References

[1] : Self-compassion In Hospice and Palliative Care. *Journal of Hospice & Palliative Nursing* .2021;23(2) : 10.1097/NJH.0000000000000727 145-154 10.1097/NJH.0000000000000727 33633095

# Response

**Published:** 2022-05-31

**Authors:** Anne Finucane

**Affiliations:** Marie Curie Cancer Care, UK

Thank you very much for the helpful comments and suggestions.  We have amended our paper in line with reviewer suggestions and will take account of these points further when writing up the results. 

  

1. We have replaced the term ‘workers’ with the term ‘staff’ so there is greater consistency throughout the paper.
2. We have added the RESTORE intervention workbook to our ISRCTN record, where further detailed on course content by week, and related exercised can be accessed: https://www.isrctn.com/ISRCTN14313559
3. Intervention content development was led by DG, with input from the wider team.  Recent doctoral research on wellbeing in palliative care staff informed much of the intervention, ^1, 2^  alongside previous research on the use of ACT to support wellbeing amongst nursing staff and psychological practitioners. ^3, 4^  We have added additional references on research that informed intervention content to the paragraph entitled ‘Content’ in the Methods section. 
4. We have added more detail and references around the rationale for including a course component on self-care for hospice, and added relevant references. Thank you for drawing our attention to this oversight.
5. As reviewer 1 points out, Module 7 is focused on self-compassion. We have added two references to support this inclusion. We agree with the reviewer that self-compassion does not come naturally to palliative care workers, and that it is important to acknowledge this in Module 8 (online LIVE workshop) when reviewing materials from the previous weeks.
6. We have corrected the typo relating to the description of ProQOL subscales, and have clarified these.
